# New insights and clinical advancements in cellular oncology

**DOI:** 10.1007/s13402-012-0083-7

**Published:** 2012-06-13

**Authors:** Ad Geurts van Kessel

**Affiliations:** Department of Human Genetics 855, Radboud University Nijmegen Medical Centre, PO Box 9101, 6500 HB Nijmegen, the Netherlands

**Keywords:** Epithelial Ovarian Cancer, Cervical Intraepithelial Neoplasia, Cellular Senescence, Senescent Cell, Oropharyngeal Cancer

## Abstract

The first joint meeting of the International Society for Cellular Oncology (ISCO) and the European Workshop on Cytogenetics and Molecular Genetics of Solid Tumors (EWCMST), organized by Bauke Ylstra, Juan Cigudosa and Nick Gilbert, was held from 4 to 8 March, 2012 in Palma de Mallorca, Spain. This meeting provided a novel and unique opportunity to jointly present the latest updates on the genetics of cancer and its implications for diagnosis, prognosis and therapy, now and in the future. Various aspects were highlighted, including the identification of effective therapeutic targets, the role of cellular senescence in tumor development and therapy, chromosome translocations in leukemias and solid tumors, mechanisms underlying fragile sites and chromosome instability, tumor-associated ‘omics’ landscapes, genetic and epidemiologic risk factors, the role of tissue and cancer stem cells, angiogenesis and the tumor micro-environment, and the epigenetics of cancer. In this report, new insights and clinical advancements related to these various topics are provided, based on information presented at the meeting.

## Targets for effective therapy

Over the last years substantial advances in the treatment of solid tumors have been made. Molecular markers, e.g. Her2/neu, KRAS, EGFR and BRAF, which can predict the therapeutic success of some monoclonal antibodies and tyrosine kinase inhibitors, are now used in clinical practice. As a result, selected patients with breast, gastro-esophageal, colorectal and non-small cell lung cancer can be treated substantially more effective.

The transcription factor MYC coordinates the expression of a vast and functionally diverse repertoire of genes that, together, are required for the orderly proliferation of somatic cells. These include genes that govern processes such as the cell division cycle and cell survival, as well as a multitude of processes that proliferating cells need to engage in their surrounding microenvironment, such as the generation of blood vessels, tissue remodeling and the recruitment of cells loaded with enzymes and growth factors needed to do this. MYC is functionally non-redundant and absolutely required for the efficient proliferation of normal and cancer cells. The control of MYC expression in cancer cells is almost always compromised, which turns MYC into a compelling candidate cancer drug target [1]. As of yet, however, the direct targeting of MYC has proven to be elusive. If MYC can’t be targeted, what can be done? One intriguing idea is that oncogenic mutations that drive cancer necessarily impose novel dependencies on other, collateral pathways [2]. Indeed, MYC triggers programmed cell death (apoptosis) that must first be blocked by cooperating oncogenic mechanisms before its oncogenic capacity can become manifest. To find such ‘synthetic lethalities’ Kessler et al. [3] very recently performed an unbiased screen using small hairpin interfering RNAs (shRNAs) to block gene expression in human mammary epithelial cells engineered to express oncogenic MYC. By doing so, they identified several factors already implicated in the survival of MYC-driven cancer cells, including MDM2 and, surprisingly, also a SUMO-activating enzyme (SAE). The inhibition of SAE triggered mitotic spindle defects in MYC expressing cells, thereby eliciting mis-segregation of chromosomes and in conjunction cell death, thus providing a proof-of-concept for this idea [4].

In his keynote lecture, Gerard Evans reported the development of a switchable genetic mouse model in which endogenous MYC function can be systematically and reversibly inhibited in normal and tumor tissue in vivo. Using this model, he showed that inhibiting MYC has indeed a remarkably efficacious and durable therapeutic impact on multiple cancer types while eliciting only mild, reversible side effects. Since the requirement of MYC cannot be circumvented, the emergence of resistant clones was not observed. Similarly, switchable genetics was used to model the therapeutic efficacy of restoring p53 gene function in various cancer types and at various stages of their evolution. These latter studies uncovered inherent limits in the efficacy of p53-restoration as a targeted cancer therapy.

## Cellular senescence and cancer

High levels of cellular stress are intrinsically associated with the process of tumorigenesis. Also, chemotherapy and cancer regression are based on the infliction of high levels of cellular stress. For years, it has been assumed that apoptosis was the relevant cellular response. Challenging this long held assumption, current accumulated evidence indicates that senescence is the most prevalent cellular response to stress, implicated in both protecting from cancer emergence and triggering cancer regression. The fate of senescent cells within tissues has, however, long been uncertain. Some show extremely lengthy residency times in vivo. For example, moles formed by senescent melanocytic cells can reside in human skin for decades [5]. Until recently, the possibility that senescent cells could be selectively targeted for elimination was generally overlooked. This changed with the discovery that such cells secrete signaling molecules that contribute to the establishment of senescence and promote tissue repair by inducing proliferation of neighboring cells and attracting cells of the immune system. Rapid clearance of senescent cells by the immune system was first observed in mouse liver cancers. In a recent study Kang et al. [6] demonstrated that the clearance of liver tumors, induced by oncogenic NRAS in the mouse, depended on CD4^+^ T-cells. They further extrapolated their findings to human disease by analyzing the livers of patients infected with hepatitis C virus (HCV). In agreement with the mouse data, they found that senescent cells were cleared in patients infected with HCV alone, but not in those infected with both HCV and HIV. Of note, HIV infection and immunosuppressant therapy are associated with an increased risk of liver cancer [7]. The reprogramming of differentiated cells into induced pluripotent stem (iPS) cells also entails cellular stress that triggers senescence. Serrano and co-workers have previously reported that the p16/Arf locus is a main barrier to this reprogramming [8]. iPS cells represent promising therapeutic tools for many diseases. However, the intrinsic self-renewal and pluripotency renders them tumorigenic, and hence, hinders their application. Very recently, Serrano and co-workers presented a novel approach to limit their tumorigenicity through the introduction of extra copies of tumor suppressors (p16/Arf or p53; [9]).

## Chromosome translocations and cancer

Chromosome translocations may occur as somatic, primary events in various forms of cancer, including leukemias, lymphomas, sarcomas and carcinomas. These aberrant chromosomes have major consequences for cancer initiating cells. Genomic studies of translocation breakpoints have revealed mechanisms involving unresolved double strand breaks and non-homologous end joining, sometimes accompanied by regional inversion of sequences at the breakpoints. Terry Rabbitts and co-workers have been interested in developing integrated technologies to evaluate the role of translocations in cancer development and to assess the therapeutic promise of translocation-encoded oncogenic proteins. To this end, mouse preclinical models that emulate chromosomal translocations have been designed (chromosome translocations à la carte). These allow the dissection of the genetic and transcriptomic steps involved from cancer initiation (translocations) to overt disease. For therapeutic target validation, they have developed a series of methods to select antibody fragments that interfere with protein-protein interactions inside cells for target validation and to gain insight into transcription factor complex formation [10]. The chronic myeloid leukemia-associated BCR-ABL fusion protein was taken as a paradigm. This protein localizes in the cytoplasm where its oncogenic signaling leads to proliferation. If forced to the nucleus, however, BCR-ABL causes apoptosis [11]. To achieve nuclear translocation, binding domains for the capture of BCR-ABL were generated and attached to proteins with signals destined for the nucleus. At least one of these constructs (4NLS-CCmut3) turned out to be effective [12]. Terry Rabbitts proposed that these methods should be applicable to study a wide range of chromosomal translocations and their gene products in human cancers.

For a long time, a common misconception has been that fusion genes such as BCR-ABL were found in leukemias, lymphomas and sarcomas, but not in common epithelial cancers. This view has changed dramatically since the discovery of TMPRSS-ERG gene fusions in ~50 % of prostate cancers [13]. Paul Edwards pointed out that a reasonable current view would be that all neoplasms have a similar mixture of fusion genes, point mutations, etc. [14]. The discovery of gene fusions in epithelial cancers has been dramatically accelerated with the introduction of new technologies such as array painting and, more recently, next generation sequencing in a ‘paired end read’ mode. By using this technology, Robinson et al. [15] identified two classes of recurrent gene fusions involving members of the MAST and NOTCH families. These gene fusions appeared to be mutually exclusive and, together, appeared to be present in up to 5-7 % of breast cancers. Since these findings may point at novel treatment options, they illustrate the power of next generation sequencing as a tool in the development of personalized medicine. The work of Paul Edwards and co-workers has shown that a typical breast carcinoma may express ten or more fusion genes, many of which look like ‘drivers’.

## Fragile sites and chromosome instability

Common fragile sites (CFSs) are large chromosomal regions prone to breakage upon replication stress, which is considered to be a driving force of oncogenesis. CFSs were long believed to contain sequences blocking replication fork progression, thus impeding replication completion and leading to DNA breaks upon chromosome condensation. Recently, Michelle Debatisse and co-workers have shown that the fragility of FRA3B and FRA16D, the most active CFSs in human lymphocytes, rather depends on the presence of large initiation-poor regions in the core of the sites [16]. This feature forces forks coming from flanking regions to cover long distances in order to complete replication, leaving the sites incompletely replicated upon fork slowing. In addition, important variations of the replication profile along FRA3B and FRA16D have been observed across different cell types. In fibroblasts, for example, numerous initiation events occur very late in the S or G2 phases all along the initiation-poor region present in lymphoblastic cells. Remarkably, FRA3B and FRA16D are modestly expressed in fibroblasts. The finding that CFS expression is cell type-dependent indicates that, in conjunction with origin usage and timing, their instability may be determined epigenetically. Michelle Debatisse and co-workers have mapped CFSs at the molecular level in human fibroblasts [17]. As expected, the CFS distribution was found to differ extensively in fibroblasts and lymphocytes. The two major sites identified in fibroblasts, located at 3q13.3 and 1p31.1, both displayed a large initiation-poor core in fibroblasts but not in lymphocytes, in which many initiation events occur in late S and G2 phases. Noticeably, these two sites are not or only weakly fragile in lymphocytes. These observations show that commitment to fragility depends on the same replication features in both lymphocytes and fibroblasts, but that different chromosomal regions are committed in each cell type. These notions definitely confirm the epigenetic nature of CFSs and the key role of initiation-poor regions in their fragility. Most CFSs mapped at the molecular level in lymphocytes host genes more than 650 kb in size, a property conserved in the two fibroblastic sites identified at 3q13.3 and 1p31.1. Furthermore, a strict correlation was found between the location of CFSs and that of very large genes in various epithelial cells. Results were obtained suggesting that origins are prevented to fire upon expression of the large genes nested in the sites. Michelle Debatisse presented a model integrating these new concepts with the long-described role of CFS instability in oncogenesis [18].

Faithful chromosome segregation is required for cell viability and relies on both the mitotic spindle assembly checkpoint and the machinery that corrects kinetochore-microtubule attachment errors [19]. Most solid tumors mis-segregate whole chromosomes, which results in a phenomenon called chromosome instability (CIN). CIN positively correlates with a poor prognosis indicating that reduced mitotic fidelity contributes to cancer progression by increasing genetic diversity among tumor cells. To determine the mechanism leading to the loss of mitotic fidelity in CIN, Duane Compton and co-workers used live cell imaging and clonal cell analyses to evaluate chromosome segregation in chromosomally stable and unstable human cells. These experiments revealed that the most common mitotic defect in human tumor cells with CIN is the persistence of erroneous kinetochore-microtubule attachments (i.e., merotely). In normal diploid cells erroneous attachments arise spontaneously and are efficiently corrected to preserve genomic stability. However, kinetochore-microtubule attachmentsx in cancer cells with CIN are inherently more stable than those in normal diploid cells. The observed differences in attachment stability account for the persistence of mal-attachments into anaphase, where they cause chromosome mis-segregation. Importantly, decreasing the stability of kinetochore-microtubule attachments by overexpression of the kinesin-13 proteins MCAK or Kif2b suppresses CIN and restores faithful chromosome segregation fidelity to aneuploid cancer cells. Together, these data indicate that cancer cells have a diminished capacity to correct erroneous kinetochore-microtubule attachments and ongoing work of Duane Compton and co-workers has revealed that there are numerous molecular targets that undermine the attachment stability of kinetochore-microtubules, including the mitotic spindle assembly checkpoint protein MAD2 [20]. Interestingly, Richarda de Voer reported that another mitotic spindle assembly checkpoint protein, BUB1, is recurrently affected by constitutional mono-allelic chromosome micro-deletions, thus resulting in a non-classical form of mosaic variegated aneuploidy (MVA), which is associated with mild congenital features and a predisposition to develop (gastrointestinal) malignancies at early adulthood [21].

Chromosome segregation errors can also result in structural chromosome aberrations. Chromosomes that mis-segregate are frequently damaged during cytokinesis, triggering a DNA double-strand break response in the respective daughter cells involving ATM, CHK2, and p53. Rita Maia and co-workers reported that these double-strand breaks can lead to unbalanced translocations in the daughter cells [22].

## Tumor-associated ‘omics’

Next generation sequencing technologies are increasingly being used to uncover the spectrum of genomic, transcriptomic and epigenomic variants present in solid tumors [23]. Susie Cooke discussed the occurrence of somatically acquired rearrangements in high-grade serous ovarian cancers (HGSOC). These cancers are characterized by genomic instability, ubiquitous p53 loss, and frequent development of platinum resistance. Loss of homologous recombination (HR) is a mutator phenotype present in ~50 % of HGSOCs and confers hypersensitivity to platinum treatment. In order to resolve the occurrence of platinum resistance, Suzie Cook and co-workers applied next generation paired-end sequencing to identify structural variants in cell lines established before and after clinical resistance had emerged. By doing so, they found frequent tandem duplications without any evidence of HR or mismatch repair deficiency. The tandem duplication mutator phenotype arose early in the progression in vivo and persisted throughout evolution both in vivo and in vitro, which may have enabled a continual evolution. This phenotype appears to be mutually exclusive with BRCA1/2 mutation carriers [24].

Neuroblastoma is a childhood tumor of the peripheral sympathetic nervous system. The pathogenesis has for a long time been quite enigmatic, as only very few gene defects were identified in this often lethal tumor. Until recently, frequently detected gene alterations included MYCN amplifications and ALK activations. Jan Molenaar and co-workers applied microarray-based mRNA profiling, qPCR-based microRNA profiling, DNA copy number profiling using aCGH and SNP arrays and, very recently, whole genome sequencing to a large series of primary neuroblastoma tumors of all stages, with the aim to identify new tumor driving aberrations that may be employed for the development of targeted therapies. The vast amount of data has been made available through a web-based platform (http://R2.amc.nl). This platform allows the integrative analysis of high throughput data in combination with publicly available data. The analysis of structural defects led to the identification of local shredding of chromosomes, known as chromothripsis, in 18 % of high-stage neuroblastomas. These tumors are associated with a poor clinical outcome. Structural alterations recurrently affected ODZ3, PTPRD and CSMD1, which are involved in neuronal growth cone stabilization. In addition, ATRX, TIAM1 and a series of regulators of the Rac/Rho pathway were found to be mutated, further implicating defects in neuritogenesis in neuroblastoma. The genomic landscape of neuroblastoma thus reveals two novel defects, chromothripsis and neuritogenesis alterations [25]. Finally, using integrative analyses, Jan Molenaar and co-workers could identify novel driving oncogenes in neuroblastoma, including LIN28B and FOXR1, and validated BIRC5 and BCL2 as potential new intervention targets.

## Risk factors for cancer

Common genetic variation has been shown to contribute to cancer, including colorectal cancer (CRC), risk. The heritable component of CRC variance is ~35 %, but <5 % of cases are attributable to known highly penetrant mutations. Our understanding of the genetic basis of CRC has moved forward considerably in recent years, and Malcolm Dunlop and co-workers recently reported the assessment of a set of single nucleotide polymorphisms (SNPs) near 157 DNA repair genes in three CRC genome wide association studies (GWAS; [26]). Although no individual SNP showed evidence of association, the set of SNPs as a whole was associated with CRC risk. Overall, the absence to date of disease-associated DNA repair SNPs in cancer GWAS may be explained by a combination of (i) many loci with individually a very small effect on risk (ii) rare alleles of moderate effect and (iii) subgroups of CRC, such as those with microsatellite instability, associated with specific variants. Much of the heritable risk of CRC appears to be attributable to the co-inheritance of multiple low-risk variants, and rigorous multi-center statistic and methodological studies need to be conducted to solve this issue. This has been the motivation for establishing the COGENT consortium, which now includes over 20 international research groups [27]. During his presentation, Malcolm Dunlop focused on recent discoveries of 20 common genetic risk variants in the context of the overall genetic architecture of colorectal cancer. The functional correlates of variation at such risk loci were discussed, as also the potential use of genetic risk profiling for cancer prevention to tailor the intensity of surveillance (or preventative approaches) to the predicted level of risk.

While the major risk factor for the development of oropharyngeal cancers is a history of smoking and drinking, there is a rapidly growing group of younger patients developing this cancer without these risk factors. This is probably due to high rates of human papillomavirus (HPV) in these cancers. To characterize the distinct molecular alterations that occur in different oropharyngeal cancers David Smith and co-workers performed RNA and whole exome sequencing and, in addition, profiled a number of oropharyngeal cancers using methlyation arrays. Tumors and matched normal tissues were analyzed from the same patients to characterize tumor-specific alterations that occurred during cancer development. Three different groups of oropharyngeal cancer patients were compared: (i) current smokers, (ii) non-smokers and (iii) ex-smokers with at least 10 years of smoking cessation. It was observed that tumors derived from the current smoking group had both much greater variability in the number and types of alterations and many more alterations than tumors from the other two groups. It was also found that after at least 10 years of smoking cessation, gene expression and methylation patterns in both matched normal tissue and oropharyngeal tumor tissue were more similar to the non-smokers than the current smokers. In addition, a group of interesting genes was found that was inactivated by hyper-methylation only in the current smoking group. These same genes showed lower expression levels in the tumors as compared to their matched normal tissues, but again only from the current smoking group. Pathway analysis revealed alterations in the expression of genes involved in the p53 DNA damage-repair pathway, including CHECK2 and ATR, which displayed patterns of increased expression that were associated with HPV-negative current smokers rather than former never smokers [28]. Currently, David Smith and co-workers are working on tools, including FusionHunter [29], to obtain a more coherent picture of the alterations that underlie the development of these cancers and the relationship between various risk factors and distinct genomic rearrangements.

## Tissue and cancer stem cells

As of yet, it is not entirely clear why targeting any specific gene results in tumor regression. One emerging concept that attempts to explain tumor regression upon the inactivation or repair of a single mutant gene product is the idea of oncogene addiction, first described by Weinstein [30]. By this paradigm, the survival of cancer cells becomes dependent on the continued activation of particular mutant oncogenes that can induce the immortalization of at least a subset of the cells. These so called cancer stem cells (CSCs) are required to sustain a neoplastic phenotype. Dean Felsher and co-workers have developed a unique experimental system to study the mechanism by which oncogenes initiate and sustain tumorigenesis. Using this system, they were able to conditionally regulate oncogene expression in vivo in a temporally controlled and tissue-specific manner. By doing so, they showed that many oncogenes (MYC, RAS, BCR-ABL) induce tumorigenesis that is completely reversible upon their inactivation. This form of oncogene addiction was found to be strongly associated with a permanent shut down of the self-renewal programs and the induction of cellular senescence. It was found that the ability of oncogene inactivation to suppress self-renewal programs was exquisitely dependent upon the coordination of both tumor cell intrinsic cell-autonomous and host-dependent cell-dependent mechanisms [31]. Upon oncogene inactivation global and permanent changes in gene expression associated with global epigenetic changes associated with cellular senescence programs were seen. In addition, the tumor cells were found to secrete autocrine factors critical to elicit changes in gene expression programs required to induce cellular senescence. The recruitment of immune cells appeared to be pivotal for oncogene inactivation to remodel the tumor microenvironment that contributes to cellular senescence to cause sustained tumor regression. Finally, Felsher and co-workers could mathematically model and predict oncogene addiction as a consequence of changes in signaling programs. Together, this work suggests that the ability to suppress the CSC program is a likely convergent mechanism of oncogene addiction and that oncogene addiction can be modeled and predicted and, thus, exploited as a strategy to treat cancer [32, 33].

Tissue stem cells are responsible for tissue homeostasis and repair and have been hypothesized to be at the origin of CSCs. However, for the vast majority of cancers the cell of origin is still unknown. Two epithelial skin cancers are frequent in human populations: basal cell carcinoma (BCC) and squamous cell carcinoma (SCC). Cédric Blanpain and co-workers developed new genetic approaches to identify the cells of origin of BCC and SCC. Using mice conditionally expressing constitutively active Smoothened mutant (SmoM2) or KRAS mutant (G12D), they were able to activate hedgehog or RAS signaling in different cellular compartments of the skin epidermis, and to determine from which epidermal compartments oncogene expression induces cancer formation. Using clonal analyses, they found that SmoM2-induced BCCs arise from long-term resident progenitor cells of the inter-follicular epidermis. In contrast, expression of mutant KRAS in hair follicle bulge stem cells, but not in their transient amplifying progeny, led to benign squamous skin tumor (papilloma). Whereas no malignant tumor was observed following KRASG12D expression alone, expression of KRASG12D combined with loss of p53 induced invasive SCC formation. These studies have uncovered the cells at the origin of SmoM2-induced BCCs and KRAS-induced SCCs in mice, and have demonstrated that the expression of differentiation markers in tumor cells is not necessary predictive for cancer initiating cells. Therefore, they developed a novel strategy to isolate, by flow cytometry, tumor initiating cells at different time points following oncogene expression and determined the molecular changes associated with tumor initiation and progression. The functional relevance of these changes in the regulation of epidermal stem cells and human skin cancer development was discussed [34].

Hematopoietic stem cells (HSCs), the best understood adult stem cells, function to sustain all blood lineages. Using HSC as a stem cell model system, Boyi Gan and co-workers have sought to understand the role of tumor suppressors involved in cancer metabolism and the regulation of stem cell homeostasis, including TSC, a key negative regulator of mTORC1 activation and cell growth, and LKB1, a critical component controlling energy metabolism [35]. They found that ablation of LKB1 in adult mice results in severe pancytopenia and subsequent lethality. Loss of LKB1 led to impaired survival and escape from quiescence of HSCs, resulting in exhaustion of the HSC pool and a marked reduction in HSC repopulation potential. The adverse impact of LKB1 deletion on hematopoiesis was predominantly cell-autonomous and mTORC-independent, thus revealing that the TSC-mTORC1-mediated cell growth machinery and the LKB1-directed energy sensing pathway regulate HSC biology via mechanistically distinct routes. Comparative transcriptomic and functional analyses revealed the PGC-1 mediated mitochondria biogenesis pathway as a potential novel effector of LKB1-directed regulation of HSC homeostasis. Together, these studies have delineated an intimate link between tumor suppressor pathways that control cancer metabolism and those that regulate stem cell homeostasis, and point to the critical importance of coupling energy availability and tissue homeostatic demands.

The formation of metastasis is a multi-step process with several rate-limiting steps. While dissemination of tumor cells appears to be an early and frequent event, successful initiation of metastatic growth, a process termed ‘metastatic colonization’, is rather inefficient for many cancer types and accomplished only by a minority of cancer cells that reach distant sites. Prevalent target sites are characteristic for many tumor entities. Ilaria Malanchi and co-workers have shown that a small population of CSCs in a mammary gland tumor model is critical for metastatic colonization. These CSCs represent a stable population of tumor cells which selectively survive and proliferate upon metastatic seeding, thereby driving the initial expansion of cancer cells at the secondary site [36].

## Angiogenesis and tumor micro-environment

Angiogenesis is known to play a pivotal role in the etiology and progression of several cancers, including epithelial ovarian cancer (EOC). In a recent phase 3 trial the effect of bevacizumab, a vascular endothelial growth factor (VEGF) inhibitor, on survival of women with this disease was examined [37]. It was found that bevacizumab improved the progression-free survival. The benefits with respect to both progression-free and overall survival were greater among those at high risk for disease progression. Despite its histological heterogeneity and phenotypic diversity, however, EOC is largely treated as a single disease. Using microarray-based expression profiling Charlie Gourley and co-workers aimed to identify novel molecular subgroups in order to entail and improve personalized therapy. Expression analyses were performed on 363 EOC samples and linked to prospectively collected clinical data. Unsupervised clustering was applied to the most variable genes and led to the identification of six subgroups, which were significantly related to histology and overall survival. Clear cell tumors were present within one group, mucinous tumors within another group, and serous and endometrioid tumors were spread across four groups each. Among the pure serous samples, three subgroups could be identified with different overall survival rates. The dominant discriminatory factor within this cohort was related to vasculature development, more specifically angiogenesis, i.e., up-regulation of angiogenesis-related genes predominated in one of the three serous subgroups. Charlie Gourley and co-workers proposed that this subgroup may explain the reported efficacy of bevacizumab in EOC [37].

Intra-vital microscopy has revealed an abnormal organization, structure and function of the tumor microvasculature (i.e., hyper-permeability, heterogeneous and compromised blood flow). These abnormalities result in elevated interstitial fluid pressure and hinder the delivery of therapeutic agents to tumors. Furthermore, they induce a hostile microenvironment in tumors characterized by hypoxia and acidosis. The abnormal microenvironment further lowers the effectiveness of anti-tumor treatment such as radiation and chemotherapy. However, one can also exploit the aberrant microenvironment for the treatment of tumors, i.e., enhanced permeability and retention effects of the tumor vasculature may allow a selective delivery of nanoparticles to tumors. Such nanoparticles not only increase the therapeutic index but also allow the targeted delivery of toxic agents and hydrophobic drugs to tumors. Until recently, however, there was a crucial drawback of this approach, i.e., these nanoparticles could not penetrate into tumor tissues after extravasation from blood vessels. Dai Fukumura and co-workers have proposed to solve this dichotomy using a multistage nanoparticle delivery system [38]. To this end, they developed a nanoparticle that can change in size upon exposure to the microenvironment by dissociating smaller nanoparticles from large core particle using enzymes uniquely present in tumor tissues. By doing so, they aimed at taming the malicious tumor microenvironment, i.e., an imbalance of production and distribution of pro- and anti-angiogenic factors might cause a derangement of tumor angiogenesis. Restoring the balance of these factors in tumors might ‘normalize’ the tumor vasculature and thus, improve its function. Indeed, administration of cytotoxic therapies during periods of vascular normalization has been shown to potentiate their efficacy. Together, the abnormal tumor microvasculature and the abnormal tumor microenvironment led to treatment resistance. Dai Fukumura and co-workers propose that novel approaches to exploit and/or tame such abnormalities may enhance the efficacy of existing and future, novel treatment strategies.

For the development of strategies distinct from targeting the VEGF signaling axis, the identification of specific markers of tumor endothelial cells and the design of high-affinity specific drugs targeting these markers, are needed. To this end, Arjan Griffioen and co-workers set out to profile the antigenic make-up of tumor endothelial cells. For the identification of tumor-specific angiogenesis markers, they performed yeast 2-hybrid screens using existing angiogenesis inhibitors as baits, in conjunction with transcriptome subtraction techniques using RNA from angiogenic endothelial cells isolated from both malignant and normal angiogenic tissues and of resting endothelial cells. By doing so, they identified a series of genes that showed specific over-expression in tumor endothelium but not in angiogenic endothelium of normal tissues, thus creating a therapeutic window for tumor vasculature specific targeting. Antibody targeting of four cell-surface expressed or secreted products (i.e., vimentin, galectin-1, HMGB1 and IGFBP7) inhibited angiogenesis in vitro and in vivo [39, 40] and targeting of endothelial vimentin or galectin-1 in a mouse tumor model significantly inhibited tumor growth and reduced micro-vessel density. These results demonstrate the utility of the identification and subsequent targeting of tumor-specific endothelial markers for anticancer therapy. Next to the targeting of these markers for direct therapeutic use, it is possible to use ligands of these markers for the targeted delivery of drugs or tracers for imaging. As an example, a therapeutic ligand of galectin-1 was successfully used for the delivery of a fluorescent dye and of a gadolinium-based tracer for visualization of tumor angiogenesis through fluorescence microscopy and magnetic resonance imaging [41]. Patrycja Nowak-Sliwinska and co-workers reported the use of anti-angiogenic compounds in conjunction with photodynamic therapy (PDT) to improve the ultimate response. Topical administration of sunitinib, sorafenib or erlotinib after PDT resulted in a significant prolongation of PDT-induced vascular occlusion, whereas topical administration of sorafenib turned out to completely inhibit the angiogenic response within PDT treated zones. These results suggest a therapeutic potential of these anti-angiogenic compounds in combination with PDT [42].

## Epigenetics of cancer

Epigenetic changes represent common and functionally important alterations that contribute to carcinogenesis [43]. Aberrant DNA methylation was first linked to cancer more than 25 years ago. Since then, many studies have associated hypermethylation of tumor suppressor genes and hypomethylation of oncogenes to tumorigenic processes. Several of these studies (conducted mainly by the ENCODE consortium) used microarray-based technologies to assess epigenetic features of representative areas of the human genome. More recently developed next-generation sequencing technologies, in conjunction with immune-precipitation, has enormously increased throughput while dramatically reducing costs. These developments have led to several human epigenome projects, including the International Human Epigenome Project, and the concomitant design of novel epigenetic therapies.

Infection with high-risk human papillomavirus (hrHPV) is a necessary but insufficient cause of cervical cancer. Progression of hrHPV-induced cervical intraepithelial neoplasia to invasive cancer is a rare event driven by (epi)genetic alterations in the host cell genome. Current developments in population-based cervical screening by primary hrHPV testing ask for objective biomarkers for risk stratification of hrHPV-positive women. Renske Steenbergen and co-workers reported on DNA methylation events associated with HPV-induced malignant transformation. Both a longitudinal in vitro model system and tissue specimens were analyzed for DNA methylation alterations using quantitative methylation-specific PCR (qMSP). Following functional studies in HPV-transformed keratinocytes, the diagnostic potential of most promising methylation markers was evaluated on hrHPV-positive cervical scrapes. An increasing number of genes was found to become methylated during hrHPV-induced carcinogenesis, including Wnt-pathway regulators, novel tumor suppressor genes, miRNAs and viral DNA. For CADM1, MAL and miRNA-124-2 DNA methylation-based gene silencing was demonstrated to functionally contribute to hrHPV-induced transformation. Based on their distinct onset during transformation and extensive evaluation on tissue specimens the combination of CADM and MAL methylation was tested on hrHPV-positive cervical scrapes collected during population-based cervical screening studies using a training and independent validation set. Testing for CADM1/MAL methylation appeared as least as discriminatory for high-grade cervical intraepithelial neoplasia as the currently proposed method, i.e. cytology either or not combined with HPV16/18 genotyping. Steenbergen and co-workers conclude that testing for DNA methylation alterations provides an objective triage tool for hrHPV-positive women. This opens up the possibility for complete cervical screening by objective, non-morphological molecular methods applicable to both conventional scrapes and self-sampled cervico-vaginal specimens.

Aberrant DNA methylation and histone modification also play an important role in the malignant transformation of hematopoietic cells. In the past years, novel mutations have been described in genes from the epigenetic machinery (TET2, DNMT3A and EZH2) as well as in metabolic genes (IDH1 and IDH2), which impact the epigenetic status of the leukemic blasts [44–47]. By using the HELP assay, a custom DNA methylation microarray, Maria Figueroa and co-workers profiled at a genome-wide scale a large cohort of primary AML and ALL samples. Through a combination of supervised and unsupervised approaches they were able to determine that (i) DNA methylation is not randomly altered, but that in fact distinct DNA methylation patterns characterize different subtypes of both AML and ALL, (ii) DNA methylation allows the identification of previously unrecognized subtypes of both these diseases, (iii) aberrant loss of promoter methylation is a much more common phenomenon than previously thought, (iv) secondary AML presents more profound epigenetic abnormalities than de novo AML, and (v) DNA methylation biomarkers can be used to successfully predict clinical outcomes. Furthermore, in both ALL and AML they observed the presence of recurrent epigenetic lesions, predominantly hyper-methylation, which appeared to affect almost all cases, irrespective of genetic or mutational subtype. These recurrent epigenetic lesions, which are likely to play cooperative roles in leukemogenesis, were specific for either AML or ALL, with only partial overlap between the two diseases, thus indicating that this epigenetic component of leukemogenesis is highly lineage specific.

## Epilogue

It is anticipated that with the continuously increasing power of cellular, functional and genetic screens, including the application of next-generation sequencing, cancer-causing genes and/or mechanisms will be elucidated at an increasingly rapid pace. This, in turn, will provide further insights into the etiology of cancer and opportunities to improve diagnosis, prognosis and (personalized) therapy.
